# Three-dimensional ultrasound assessment of risk factors for cystocele and Green classification in primipara

**DOI:** 10.3389/fmed.2022.979989

**Published:** 2022-11-30

**Authors:** Weiwei Yin, Qianqing Ma, Wen Xie, Yuting Zhu, Junli Wang

**Affiliations:** ^1^Department of Ultrasound, Second People’s Hospital of Wuhu, Wuhu, Anhui, China; ^2^Department of Ultrasound, The First Affiliated Hospital of Anhui Medical University, Hefei, Anhui, China

**Keywords:** ultrasound, vaginal delivery, cystocele, Green classification, pelvic floor dysfunction

## Abstract

**Background and aims:**

The present study aimed to analyze the effects of factors on cystocele and the Green classification.

**Materials and methods:**

We conducted a cross-sectional study on 357 primiparous women examined at our hospital from January 2019 to May 2021. The following data were recorded: maternal characteristics, neonatal characteristics, and factors of childbirth. It was added to the multivariate logistic regression model to determine the independent predictors of the cystocele and the Green classification.

**Results:**

A total of 242 women had cystocele, including 71 women with Green type I cystocele, 134 women with Green type II cystocele, and 37 women with Green type III cystocele. In multivariate logistic regression analysis, body mass index (BMI) at delivery was associated with cystocele, while BMI at delivery and the second stage of labor (SSL) > 1 h were independently with the distance from the symphysis pubis to the bladder neck (SPBN) abnormal (*P* < 0.05). BMI at examination was associated with the large retrovesical angle (RVA) (*P* < 0.05). BMI at delivery and the fetal right occiput anterior position (ROA) were independently associated with the distance from the symphysis pubis to the posterior wall of the bladder (SPBP) abnormal (*P* < 0.05), while epidural anesthesia (EDA) was the protective factor (*P* < 0.05).

**Conclusion:**

Primipara women should strive to avoid exposure to modifiable risk factors such as controlling weight during pregnancy, reducing weight after delivery, and shortening SSL to reduce the occurrence of cystocele.

## Introduction

Pelvic floor dysfunction (PFD) refers to a group of disorders caused by structural defects or degradation, damage, and dysfunction of the pelvic floor support system that severely affects the quality of life. Multiple symptoms of PFD may include stress urinary incontinence (SUI), pelvic organ prolapse (POP), chronic pelvic pain, constipation, and sexual abnormalities (1). Vaginal delivery (VD) significantly increases the possibility of POP in women (2), and it is the most critical epidemiological risk factor for PFD. Most of the damage to a woman’s pelvic floor is likely to occur during the first vaginal birth (3, 4). At present, most studies focus mainly on levator ani muscle (LAM) injury, but there are fewer studies on cystocele and the Green classification. Cystocele is the anterior vaginal wall prolapse accompanied by prolapse of the bladder wall, and it is the most common type of POP and the most prone to recur after surgery (5, 6). The current imaging classification of cystocele is proposed by Green. Cystocele has different clinical manifestations according to the Green’s classification (6). Radiological cystocele type (Green classification) can be distinguished both clinically and on ultrasound, and agreement between methods as well as inter-observer agreement for the clinical diagnosis is moderate to good (7).

In this study, We aim to use three-dimensional ultrasound to diagnosis cystocele and its classification, and analyzed the effects of maternal characteristics, fetal characteristics, and delivery factors on cystocele and its classification. We hypothesized that BMI after 3 months of vaginal delivery will be effect of cystocele and the Green classification.

We used three-dimensional ultrasound to detect cystocele and its type and analyzed the effects of maternal, fetal, and delivery factors on cystocele occurrence and its classification. The objective was to reduce pelvic floor damage in primipara women during childbirth.

## Materials and methods

### Study design

This was a cross-sectional study of healthy pregnant Chinese women. Details on maternal characteristics and delivery outcomes were obtained from the medical records of the our hospital from January 2019 to May 2021.

The inclusion criteria were nulliparous women with maternal age of ≥18 years, and time of ultrasonography was 42 to 90 days after delivery. Exclusion criteria were delivery at <28 gestational weeks and pre-existing diseases/conditions that are likely to pre-dispose to PFD. This included previous bladder/bowel diseases and chronic kidney disease. Participants did not perform any rehabilitation exercises. The Institutional Review Board approved the study protocol.

### Clinical data

The hospital medical record system was checked, and the following details were recorded: maternal characteristics (include maternal age, body mass index (BMI) at delivery, BMI at ultrasound examination, gestational age, hypertension, diabetes), neonatal characteristics (include fetal height, head circumference, chest circumference, birth weight), and factors of childbirth (SSL, episiotomy, perineal tear, forceps use, vacuum use, and fetal orientation).

### Ultrasonographic data

An ultrasonographic evaluation was performed by a single examiner with more than 5 years of experience in obstetric ultrasound and with specific training in 3/4D imaging. Women were examined with *trans*-perineal ultrasound using the GE Voluson E8 system with a 3D/4D RIC 5–9-D probe with an acquisition angle of 180°.

Before the examination, the subject emptied the bladder (urine volume < 50 ml) and rectum and assumed the lithotomy position. The probe was wrapped in a condom and placed above the labia minora. The probe was placed close to the lower edge of the pubic symphysis for a clear display of the midsagittal section of the pelvic floor structure and the position and shape of the bladder were observed under the resting state and Valsalva. Valsalva movement requirements: the duration lasts more than 5 s, while the levator ani muscle hiatus is observed to be dilated, and the pelvic organs are displaced to the dorsal caudal side. The lower edge of the symphysis pubis (SP) was used as the reference level to analyzed the following parameters: the retrovesical angle (RVA), urethral inclination angle, distance from the inferior margin of the symphysis pubis to the bladder neck (SPBN), and the distance from the inferior margin of the symphysis pubis to the posterior wall of the bladder (SPBP) at rest and Valsalva. The urethral rotation angle (URA) was also calculated.

A cystocele was diagnosed on ultrasound if any part of the bladder below the symphysis pubis (8). According to Green classification, the following types of cystocele were diagnosed: Green I cystocele (RVA ≥ 140°, URA < 45°), Green II cystocele (RVA ≥ 140°, URA between 45° and 120°, and Green III (RVA < 140°, the lowest point of the bladder reaching below the symphysis pubis) (9). In Green III cystocele, the lowest point of the bladder is often the posterior wall of the bladder, unlike the bladder neck in other types.

### Statistical analyses

Statistical analyses were performed using SPSS v. 20 (SPSS, Chicago, IL, USA). The normality of data was assessed using the Shapiro Wilk method. Normally and non-normally distributed continuous data were expressed as mean ± standard deviation and quartile, respectively. Normally and non-normally distributed continuous data were analyzed by Student’s *t*-test and Mann-Whitney *U* test, respectively. Categorical variables were analyzed by the chi-square test. *P* < 0.05 was considered to be statistically significant. Logistic regression analysis was used to demonstrate independent risk factors for cystocele while controlling for potential confounding factors.

## Results

A total of 357 women who had vaginal delivery were enrolled in this study. Of these, 242 (67.8%) women had cystocele, including 71 (19.9%) women with Green type I cystocele, 134 (37.5%) women with Green type II cystocele, and 37 (10.4%) women with Green type III cystocele. Moreover, 281 (78.7%) women had abnormal SPBN, 198 (55.5%) women had increased URA, 240 (67.2%) women had open RVA, and 97 (27.2%) women had abnormal SPBP.

The results for the hospital parameters were as follows: episiotomy: 93 (26.1%) women; second-degree perineal tear: 213 (59.3%) women; epidural anesthesia: 18 (47.1%) women, diabetes: 28 (7.8%) women; hypertension: 4 (1.1%) women; assisted labor: 4 (1.1%) women [including 1 (0.3%) woman with forceps-assisted delivery and 3 (0.8%) women with vacuum-assisted delivery]. The following results were noted for fetal orientation: fetal left occiput anterior (LOA) position in 252 (70.6%) women, fetal right occiput anterior (ROA) position in 81 (22.7%) women, and other fetal positions in 24 (6.72%) women [including occipital posterior (OP) position in 11 (3.1%) women and occipital bone is directly in front of the pubic symphysis position in 13 (3.6%) women].

In the univariate analysis, BMI at delivery, BMI at the examination, and birth weight in patients with cystocele were greater than those in the normal group, and the SSL in these patients was also had an impact on the cystocele group. BMI at delivery, BMI at the examination, and birth weight were greater in the abnormal SPBN group than in the normal group. SSL and different fetal positions also had an impact on SPBN (Table 1). BMI at delivery with increased URA was greater than that in the normal group. BMI at delivery, BMI at the examination, and birth weight were greater in patients with large RVA than in the normal group. The BMI of patients with abnormal SPBP was also higher than that of the normal group. Epidural anesthesia (EDA) and fetal position were found to affect SPBP (Table 2).

**TABLE 1:** Analysis of factors associated with cystocele, symphysis pubis to the bladder neck (SPBN), and urethral rotation angle (URA).

Variables	Cystocele		*P*	SPBN		*P*	URA		*P*
					
	No cystocele	Cystocele		Normal	Abnormal		Normal	Abnormal	
**Maternal characteristics**									
Maternal age (years)*	27 (25.25∼29)	28 (26∼30)	0.268^a^	27 (25∼29)	28 (26∼30)	0.177^a^	27 (26∼29)	28 (25∼30)	0.626^a^
Gestational age (weeks)*	39.5 (39∼40.2)	39.5 (39∼40.3)	0.912^b^	39.5 (39∼40.2)	39.5 (39∼40.3)	0.681^b^	39.5 (39∼40.3)	39.5 (39∼40.3)	0.685^b^
Gestational diabetes, *n* (%)	8 (2.2)	20 (5.6)	0.946^b^	5 (1.4)	23 (6.4)	0.644 ^b^	11 (3.1)	17 (4.8)	0.560^b^
Hypertension, *n* (%)	1 (0.3)	3 (0.8)	1.000^d^	1 (0.3)	3 (0.8)	1.000 ^e^	1 (0.3)	3 (0.8)	0.776^d^
BMI at delivery (kg/m^2^)*	24.82 (23.19∼26.77)	26.24 (24.61∼28.2)	**<0.001** ^a^	24.82 (23.36∼26.77)	26.13 (24.54∼28.02)	**0.002** ^a^	25.65 (23.63∼27.68)	26.15 (24.54∼27.77)	0.130^a^
BMI at examination (kg/m^2^)*	21.9 (20.69∼24.15)	23.53 (21.88∼25.49)	**<0.001** ^a^	21.9 (20.69∼24.21)	23.44 (21.59∼25.39)	**0.001** ^a^	22.58 (20.96∼24.8)	23.44 (21.7∼25.27)	**0.047** ^a^
**Neonatal characteristics**									
Fetal height (cm)*	50 (50∼50)	50 (50∼51)	0.091^a^	50 (50∼50)	50 (50∼51)	0.141^a^	50 (50∼51)	50 (50∼50)	0.740^a^
Head circumference (cm)*	34 (33∼34)	34 (33∼34)	0.829^a^	34 (33∼34)	34 (33∼34)	0.424^a^	34 (33∼34)	34 (33∼34)	0.590^a^
Chest circumference (cm)*	32 (32∼33)	32 (32∼33)	0.738^a^	32 (32∼33)	32 (32∼33)	0.358^a^	32 (32∼33)	32 (32∼33)	0.657^a^
Birth weight (g)^#^	3216.15 ± 352.28	3328.42 ± 355.47	**0.007** ^c^	3220.39 ± 343.96	3316.09 ± 359.23	**0.038** ^c^	3315.85 ± 367.66	3279.55 ± 349.61	0.341^c^
**Delivery characteristics**									
SSL < 1 h, *n* (%)	88 (24.6)	157 (44)	**0.048**	64 (17.9)	181 (50.7)	**< 0.001^f^**	113 (31.7)	132 (37)	0.581
SSL > 1 h, *n* (%)	21 (5.9)	74 (20.7)		7 (2)	88 (24.6)		38 (10.6)	57 (16)	
SSL > 2 h, *n* (%)	6 (1.7)	11 (3.1)		5 (1.4)	12 (3.4)		8 (2.2)	9 (2.5)	
EDA, *n* (%)	45 (12.6)	123 (34.5)	0.358^b^	32 (9)	136 (38.1)	0.329^b^	77 (21.6)	91 (25.5)	0.642^b^
Episiotomy, *n* (%)	27 (7.6)	66 (18.5)	0.980^b^	20 (5.6)	73 (20.4)	0.953^b^	39 (10.9)	54 (15.1)	0.557^b^
Second-degree vaginal tear, *n* (%)	62 (17.4)	151 (42.3)	0.990^b^	46 (12.9)	167 (46.8)	0.863^b^	101 (28.3)	112 (31.4)	0.183^b^
Forceps and vacuum assisted delivery, *n* (%)	1 (0.3)	3 (0.8)	1.000^d^	1 (0.3)	3 (0.8)	1.000^e^	2 (0.6)	2 (0.6)	1.000^d^
Fetal position			0.160^b^			**0.035** ^b^			0.092^b^
LOA, *n* (%)	71 (19.9)	181 (50.7)		52 (14.6)	200 (56)		115 (32.2)	137 (38.4)	
ROA, *n* (%)	29 (8.1)	52 (14.6)		23 (6.4)	58 (16.2)		37 (10.4)	44 (12.3)	
Others, *n* (%)	4 (1.1)	20 (5.6)		1 (0.3)	23 (6.4)		7 (2)	17 (4.8)	

BMI, body mass index; SSL, second stage of labor; EDA, Epidural anesthesia; LOA, left occiput anterior; ROA, right occiput anterior; SPBN, symphysis pubis to the bladder neck; URA, urethral rotation angle. Bold values are *P* < 0.05 and are statistically significant.

*Median (Inter-Quartile Range).

^#^Mean ± Standard Error.

^a^Mann-Whitney U test.

^b^Chi-square test.

^c^Student’s t-test.

^d^Continuity Correction.

^e^Fisher’s exact test.

^f^Likelihood ratio test.

**TABLE 2:** Analysis of factors associated with retrovesical angle (RVA) and symphysis pubis to the posterior wall of the bladder (SPBP).

Variables	RVA	Abnormal	*P*	SPBP	Abnormal	*P*
				
	Normal			Normal		
**Maternal characteristics**						
Maternal age (years)*	27 (25∼29)	28 (26∼30)	0.664^a^	27.5 (26∼29)	28 (25∼30)	0.724^a^
Gestational age (weeks)*	39.5 (39∼40.25)	39.6 (39∼40.3)	0.732^b^	39.5 (39∼40.3)	39.6 (39∼40.3)	0.341^b^
Gestational diabetes, *n* (%)	8 (2.2)	20 (5.6)	0.622^b^	21 (5.9)	7 (2)	0.788^b^
Hypertension, *n* (%)	0 (0)	4 (1.1)	0.385^c^	4 (1.1)	0 (0)	0.507^c^
BMI at delivery (kg/m^2^)*	25.89 (23.88∼27.24)	26.04 (24.44∼28.03)	0.094^a^	25.71 (23.86∼27.71)	26.35 (24.79∼27.84)	**0.030^a^**
BMI at examination (kg/m^2^)*	22.45 (20.7∼24.41)	23.43 (21.68∼25.44)	**0.012^a^**	23.14 (21.22∼25.24)	23.42 (21.6∼25.11)	0.374^a^
**Neonatal characteristics**						
Fetal height (cm)*	34 (33∼34)	34 (33∼34)	0.050^a^	50 (50∼51)	50 (50∼50)	0.937^a^
Head circumference (cm)*	34 (33∼34)	34 (33∼34)	0.413^a^	34 (33∼34)	34 (33∼34)	0.968^a^
Chest circumference (cm)*	32 (32∼33)	32 (32∼33)	0.777^a^	32 (32∼33)	32 (32∼33)	0.498^a^
Birth weight (g)*	3,250 (3,005∼3,500)	3,330 (3,100∼3,547.5)	0.054^a^	3,300 (3,080∼3,512.5)	3,330 (3,020∼3,545)	0.423^a^
**Delivery characteristics**						
SSL < 1 h, *n* (%)	88 (24.6)	157 (44)	0.164	183 (51.3)	62 (17.4)	0.381^d^
SSL > 1 h, *n* (%)	24 (6.7)	71 (19.9)		64 (17.9)	31 (8.7)	
SSL > 2 h, *n* (%)	5 (1.4)	12 (3.4)		13 (3.6)	4 (1.1)	
EDA, *n* (%)	50 (14)	118 (33.1)	0.253^b^	131 (36.7)	37 (10.4)	**0.039^b^**
Episiotomy, *n* (%)	30 (8.4)	63 (17.6)	0.902^b^	65 (18.2)	28 (7.8)	0.459^b^
Second-degree vaginal tear, *n* (%)	69 (19.3)	144 (40.3)	0.853^b^	160 (44.8)	53 (14.8)	0.237^b^
Forceps-assisted and vacuum-assisted delivery, *n* (%)	2 (0.6)	2 (0.6)	0.839^c^	2 (0.6)	2 (0.6)	0.640^c^
Fetal position			0.476^b^			**0.046^b^**
LOA, *n* (%)	79 (22.1)	173 (48.5)		193 (54.1)	59 (16.5)	
ROA, *n* (%)	31 (8.7)	50 (14)		52 (14.6)	29 (8.1)	
Others, *n* (%)	7 (2)	17 (4.8)		15 (4.2)	9 (2.5)	

Bold values are *P* < 0.05 and are statistically significant.

*Median (IQR).

^#^Mean ± SD.

^a^Mann-Whitney U test.

^b^Chi-square test.

^c^Continuity Correction.

^d^Likelihood ratio test.

**FIGURE 1:**
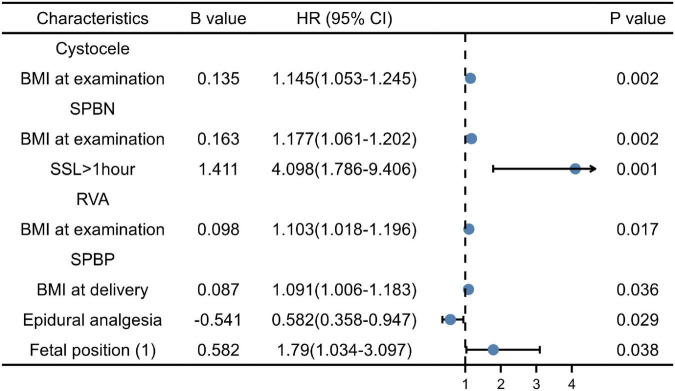
Multiple factor analysis of cystocele, symphysis pubis to the bladder neck (SPBN), retrovesical angle (RVA), and symphysis pubis to the posterior wall of the bladder (SPBP).

According to the results of the univariate analysis, the above factors were entered into the multivariate logistic regression model (forward, conditional). BMI at delivery was an independent risk factor of cystocele (OR = 1.145, 95% CI: 1.053∼1.245); BMI at delivery (OR = 1.18, 95% CI: 1.065∼1.307) was independent risk factors of SPBN (*P* < 0.05). The SSL > 1 h compared to the SSL < 1 h (OR = 4.10, 95% CI: 1.786∼1.562) was the risk factors of SPBN (*P* < 0.05). BMI at examination (OR = 1.103, 95% CI: 1.018∼1.196) was the risk factor for the large RVA (*P* < 0.05). BMI at delivery (OR = 1.091, 95% CI: 1.006∼1.183) and the fetal ROA position compared to the LOA were the risk factors of SPBP (*P* < 0.05), while EDA (OR = 0.582, 95% CI: 0.358∼0.947) was the protective factor (*P* < 0.05). In the logistic regression analysis, all factors had no significant association with URA (*P* > 0.05) (Figure 1).

## Discussion

Mechanical injury to the pelvic floor support system, denervation, ischemia and reperfusion injury, and defective soft tissue remodeling are some of the underlying mechanisms of injury for the development of PFDs (10). During pregnancy and after VD, pelvic organ support changes, and area of levator hiatus (HA) increases; thus, indicating a decrease in pelvic organ support (11, 12), This may increase the risk of PFD. The present study found that many factors influence the development of maternal cystocele during delivery.

### Maternal and neonatal characteristics

Similar to previous studies, we found that the increase in BMI at delivery was significantly associated with abnormal SPBP (OR = 1.091, 95% CI: 1.006∼1.183). Not only does BMI during pregnancy increase the risk of pelvic floor disease, but BMI after delivery also affects the occurrence of cystocele. The increase in BMI at examination was significantly associated with abnormal SPBN (OR = 1.18, 95% CI: 1.065∼1.307) and open RVA (OR = 1.103, 95% CI: 1.018∼1.196). An obvious potential explanation for the increased prevalence of cystocele in obese women may be the long-term increase in intra-abdominal pressure in these individuals. Noblett et al. (13) found a strong correlation between BMI and intra-abdominal pressure, and between BMI and intravesical pressure, with Pearson’s correlation coefficient of 0.76 and 0.71, respectively. The chronic increased pressure may lead to pelvic floor muscle fatigue and/or a chronic stretch on the pudendal nerve, which in turn may lead to pelvic floor muscle weakness. Although the decrease in pelvic muscle strength after VD will lead to POP, the correlation between them will weaken after a reduction in BMI (14). For primipara women, early weight loss can also help to reduce cystocele.

In other studies on the pelvic floor, age was found to be an important factor for cystocele (15). In the present study, no significant association was found between age and Green’s classification of cystocele. This may be because all the primipara women in this study were young. In addition, there were fewer cases of gestational diabetes mellitus and gestational hypertension in this study, neither hypertension nor GDM showed a statistically significant association with cystocele.

The most important risk factor for avulsion injuries during natural delivery is the birth weight (16). The increase in birth weight has an important effect on the time of labor and subsequently has a series of effects on the pelvic floor muscles. In our study, it had no significant in the multivariate binary logistic analysis on cystocele, only appeared as a significant variable in the Mann-Whitney *U* test.

### Delivery characteristics

Prolonged labor may exceed the stretch limit of soft tissues, resulting in an imbalance in the repair and degradation process. When used for a long time, stress may cause temporary or permanent physical and/or functional damage through hypoxia, ischemia, and other harmful processes, leading to PFD (17). We found that SSL > 1 h was 4.10 times higher than SSL < 1 h for primary maternal of abnormal SPBN. Reports on the effects of episiotomy and perineal tear on pelvic floor function are not completely consistent (18, 19). But most studies believe that the use of episiotomy during vacuum-assisted delivery or forceps-assisted delivery of primipara women can reduce perineal tear or OASIS (20, 21). We did not confirm that episiotomy and tear have a significant effect on cystocele, which is consistent with the report of Ruan et al. (22). This may be because of the low episiotomy rate or the short observation period, or it may be because of only first-and second-degree tears in our study. Studies have shown that third-degree perineal tear is a significant risk factor for postpartum PFD (23).

Schiessl B (24) found that EDA can prolong SSL, thereby increasing the risk of UI; however, in our study we think that EDA is a protective factor of SPBP (OR = 0.582, 95% CI: 0.358∼0.947). The EDA effect could be explained by the resulting muscle relaxation. It is plausible that active pushing in labor distends and compresses the pelvic floor more forcefully, resulting in neuromuscular or vascular injury. Intrapartum epidural analgesia may be beneficial by preventing premature pushing. Another potential explanation may be the degree of levator relaxation in women with dense epidurals because a paralyzed muscle is less likely to suffer trauma, given a certain degree of distension (25).

The occipital position (OP) is the most common malposition with a prevalence of 5–13% at delivery (26). It increases the risk of LAM injury (27). In the present study, there is no difference in statistics due to the low numbers of OP. However, to our surprise, the results showed that ROA was more prone to SPBP abnormalities than LOA (OR = 1.79, 95% CI: 1.034∼3.097).

### Strengths and limitations

All women underwent delivery in the same hospital following similar obstetric approaches. Moreover, all parturients had complete delivery information, which largely reduced the recall bias. Previous studies on postpartum pelvic floor injury mostly focused on factors such as birth weight, weight gain during pregnancy, and the labor process. In our study, we found that not only BMI at delivery has an important effect on cystocele in primipara women, but BMI within 42∼90 days postpartum is also an independent risk factor for abnormal SPBP and open RVA. Early postpartum weight loss can reduce pelvic floor injury.

The limitations of our study are a small sample size and relatively inadequate data. In this study, all primipara women were younger, with less degree of hypertension and diabetes. However, in future research, we will collect more samples for a more comprehensive analysis.

## Conclusion

During delivery, the pelvic floor muscles of pregnant women will continue to be affected by mechanical injury and physiological changes after delivery. Some potential risk factors are uncontrollable, such as maternal age, fetal position, and fetal weight. However, according to the currently available data, efforts should be made to avoid exposure to modifiable risk factors, such as controlling weight during pregnancy, reducing weight after delivery, shortening the SSL, and reducing the occurrence of cystocele.

## Data availability statement

The original contributions presented in this study are included in the article/supplementary material, further inquiries can be directed to the corresponding author.

## Author contributions

WY: project development, manuscript writing, data analysis, and data collection. QM: project development, data analysis, and manuscript editing. WX: data collection and manuscript editing. YZ: data analysis. JW: project development, data collection, and manuscript editing. YZ: statistics and writing of the manuscript. All authors contributed to the article and approved the submitted version.
